# Surgical outcome after thyroidectomy due to Graves’ disease and Lugol iodine treatment: a retrospective register-based cohort study

**DOI:** 10.1007/s12020-024-03708-4

**Published:** 2024-02-02

**Authors:** Fredric Hedberg, Henrik Falhammar, Jan Calissendorff, Robert Bränström

**Affiliations:** 1 Department of Endocrinology, Karolinska University Hospital, Stockholm, Sweden; 2 Department of Molecular Medicine and Surgery, Karolinska Institutet, Stockholm, Sweden; 3 Department of Breast, Endocrine Tumors and Sarcoma, Karolinska University Hospital, Stockholm, Sweden

**Keywords:** Hypocalcemia, Thyroidectomy, Graves’ disease, Lugol iodine, Short-time outcome

## Abstract

**Purpose:**

This study aimed to investigate the relationship between Lugol iodine treatment in a rescue setting and surgical outcomes in Graves’ disease patients.

**Methods:**

The retrospective register-based cohort study included 813 patients who had undergone primary total thyroidectomy with a primary diagnosis of Graves’ disease (ICD-code E05.0) at Karolinska University Hospital in Stockholm, Sweden, between January 2008 and December 2015. Of 813 patients, 33 (4.1%) were given Lugol iodine before surgery and the remaining, the non-Lugol group, did not. The study’s primary outcomes were post-operative calcium treatment day 1, calcium and vitamin D supplements at discharge and follow-up. Secondary outcomes were laryngeal nerve damage and bleeding (defined as re-operation).

**Results:**

Differences were found between the Lugol and non-Lugol groups in the treatment of calcium day 1 (45.5% vs 26.7%, *p* = 0.018), at discharge (36.4% vs. 16.2%, *p* = 0.002) and vitamin D supplements at discharge (36.4% vs. 19.1%, *p* = 0.015) as surrogate variables for hypocalcemia post-operatively. No differences could be seen at 4–6 weeks and six-months follow-up. There were no differences between the Lugol and non-Lugol groups in terms of operation time, laryngeal nerve damage, and bleeding.

**Conclusion:**

Patients in our cohort undergoing thyroidectomy due to Graves’ disease pre-operatively treated with Lugol iodine as a rescue therapy had a higher risk of experiencing short term post-operative hypocalcemia.

## Introduction

Thyroid hormones are crucial for regular metabolic activity and regulation of physiological functions in the body. However, when there is excessive production of thyroid hormones, it results in a condition known as thyrotoxicosis [1]. Various factors, including autoimmune disease, thyroid nodules, and excessive iodine intake can cause this condition. The clinical manifestations of thyrotoxicosis are diverse and can affect multiple systems, such as the cardiovascular and neuromuscular systems [1]. The treatment options include antithyroid drugs, radioactive iodine therapy, and surgery [2]. Each treatment modality has its advantages and disadvantages, and the choice of treatment depends on the underlying cause, the severity of symptoms, as well as the patient’s and treating physician’s preference. Antithyroid drugs such as methimazole and propylthiouracil are the first-line therapy in Europe for thyrotoxicosis caused by Graves’ disease [3]. Thyroidectomy is an option for patients with large goiters, suspicious nodules, or those who prefer a definitive treatment [4]. However, surgery carries the risk of complications such as bleeding, infection, and injury to the recurrent laryngeal nerve and parathyroid glands [5]. Radioactive iodine therapy is also a definite cure when applied in patients with Graves’ disease [6].

Lugol’s solution, also known as Lugol iodine, is a solution of iodine and potassium iodide in water. It is named after the French physician Jean Lugol, who first described it in 1829 [7]. Lugol’s solution, or iodine tablets, can be used as a short-term treatment to reduce the production of thyroid hormones [8]. It works by temporarily inhibiting the uptake of iodine by the thyroid gland, which in turn reduces the synthesis and release of thyroid hormones [9, 10]. According to American Thyroid Association guidelines, Lugol’s solution is recommended to most patients in preparation for thyroid surgery for hyperthyroidism caused by Graves’ disease [2], but the evidence for its efficacy in reducing surgical complications is still uncertain [11]. In our setting Lugol’s solution is only advocated when antithyroid drugs are not tolerated, the patient has not reached euthyroidism on anti-thyroid drugs, or when a rapid block of excess hormone levels is warranted [12].

By exploring the relationship between Lugol iodine treatment in a rescue setting and surgical outcomes in Graves’ disease patients, this study aims to address knowledge gap and provide valuable insights for clinicians and researchers seeking to improve the safety and efficacy of thyroidectomy procedures.

## Materials and methods

**Fig. 1:**
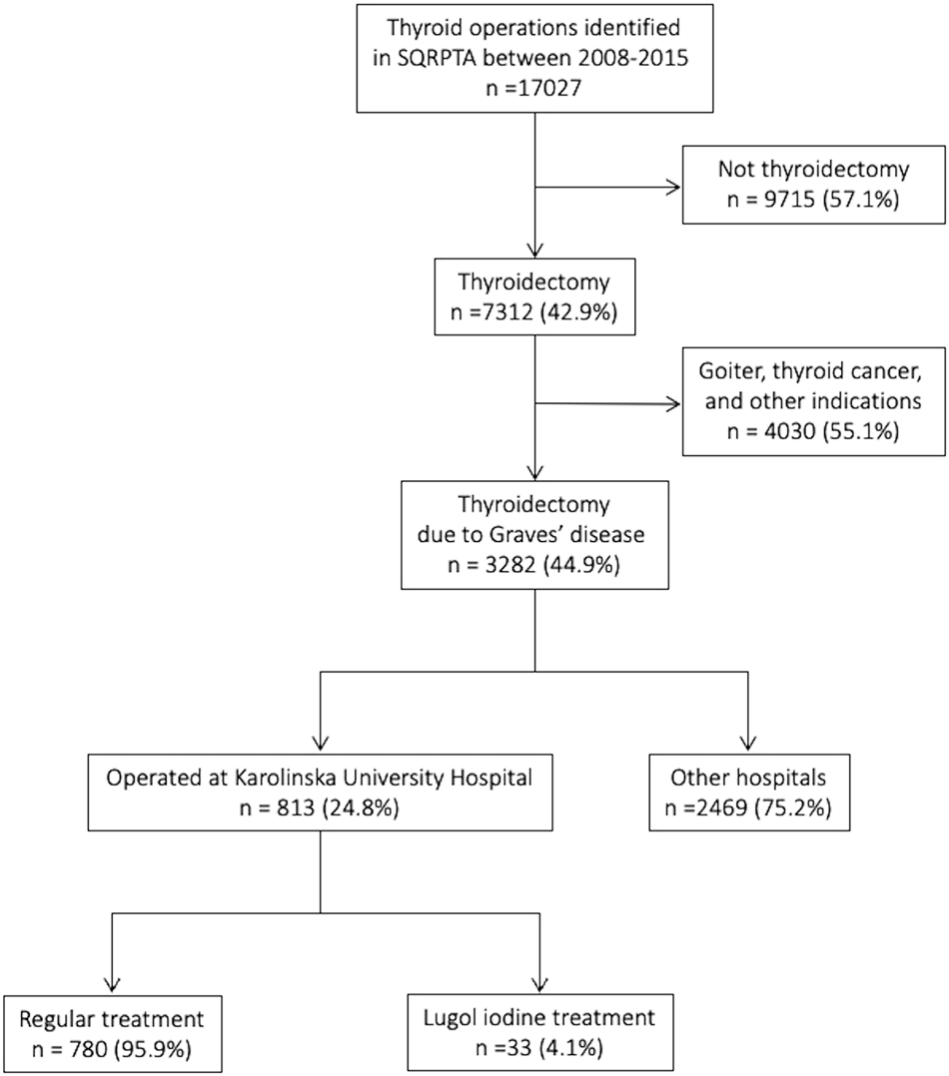
Flow-chart of the participants enrolled in this study. SQRTPA, Scandinavian Quality Register for Thyroid, Parathyroid and Adrenal Surgery

We performed a retrospective register-based cohort study on patients who had undergone total thyroidectomy from the 1st of January 2008 until the 31st of December 2015 at Karolinska University Hospital in Stockholm, Sweden. The study population was extracted from the Scandinavian Quality Register for Thyroid, Parathyroid, and Adrenal Surgery (SQRTPA) as shown in Fig. 1. All patients who had undergone primary total thyroidectomy with the primary diagnosis Graves’ disease (ICD-code E05.0) were included. SQRTPA was established in 2004 to register thyroid, parathyroid, and adrenal surgery. During this study period, SQRTPA had good coverage in Sweden and was continuously evaluated to ensure data quality. The coverage of Swedish thyroid surgery in 2015 was 87%, and almost 100% for Karolinska University Hospital in Stockholm [13]. The cohort was divided into those with preoperative Lugol use and those who had not used Lugol iodine. The indications for Lugol treatment in our setting were intolerance to anti-thyroid drugs, insufficient effect of anti-thyroid drugs, or when a rapid block of excess hormone levels was warranted.

Data on patients operated during the period were retrieved from the registry. These included pre-operative treatments (Lugol iodine, anti-thyroid drugs, beta-blockers, glucocorticoids and levothyroxine), age, sex, date of surgery, indication for surgery, type of surgery, oral and intravenous calcium treatment post-operatively, prescribed supplement vitamin D and calcium at discharge, 4–6 weeks postoperatively, and at six-month follow-up. The primary outcome in this study was post-operative calcium and vitamin D supplements, defined as having received calcium orally or intravenously immediately postoperatively up to day 1, calcium and vitamin D treatment at discharge, and 4–6 weeks and at six months follow-up. Secondary outcomes were laryngeal nerve damage, and bleeding (defined as re-operation).

### Statistics

All statistical analyses were performed using Stata/BE version 17.0. The chi^2^-test was performed on categorical variables, including post-operative calcium treatment and prescribed supplements. Categorical variables were reported as percentages. Student’s *t* test was used on normally distributed continuous data, and Mann–Whitney U test for skewed data (thyroid weight). A *p* value of less than 0.05 was considered significant.

Missing data less than five percent were deemed ignorable and outcomes were calculated using available cases.

## Results

### Patient characteristics

This study involved 813 patients, of which 33 (4.1%) were given Lugol iodine before surgery. Of the 813 patients, most were women (*n* = 716; 88.1%). No significant differences were observed between the Lugol and non-Lugol groups in terms of age, sex, or glucocorticoid treatment. However, a higher proportion of patients in the non-Lugol group were treated with anti-thyroid drugs before surgery (87.3% vs. 60.6%, *p* < 0.001), as well as pre-operative levothyroxine (80.3% vs. 51.5%, p < 0.001). On the other hand, in the Lugol group, there was a higher proportion of patients treated with beta-blockers before their surgery (57.6% vs. 31.9%, *p* = 0.002) (Table 1).

**Table 1:** Demographic data and pre-operative medication of patients diagnosed with Graves’ disease operated with thyroidectomy at Karolinska University Hospital between 2008 and 2015

		All treatments		Non-Lugol treatment		Lugol treatment		*P* value
*N (%)*		813	(100%)	780	(95.9%)	33	(4.1%)	
Female n (%)		716	(88.1%)	688	(88.2%)	28	(84.8%)	0.56
Age at operation year (±SD)	All	38.2	(±13.6)	38.2	(±13.6)	37.5	(±13.7)	0.77
	Female	37.8	(±13.4)	37.9	(±13.4)	36.6	(±13.2)	0.62
	Male	40.5	(±15.3)	40.4	(±15.3)	42.4	(±16.9)	0.78
Pre-operative anti-thyroid treatment n (%)	All	701	(86.2%)	681	(87.3%)	20	(60.6%)	<0.001
	Female	616	(86.0%)	600	(87.2%)	16	(57.1%)	<0.001
	Male	85	(87.6%)	81	(88.0%)	4	(80.0%)	0.60
Pre-operative β-blockade n (%)	All	268	(33.0%)	249	(31.9%)	19	(57.6%)	0.002
	Female	251	(35.1%)	233	(33.9%)	18	(64.3%)	0.001
	Male	17	(17.5%)	16	(17.4%)	1	(20.0%)	0.88
Pre-operative levothyroxine n (%)	All	643	(79.1%)	626	(80.3%)	17	(51.5%)	<0.001
	Female	566	(79.0%)	552	(80.2%)	14	(50.0%)	<0.001
	Male	77	(80.0%)	74	(80.4%)	3	(60.0%)	0.27
Pre-operative glucocorticoids n (%)	All	55	(6.8%)	53	(6.8%)	2	(6.1%)	0.87
	Female	43	(6.0%)	41	(6.0%)	2	(7.1%)	0.80
	Male	12	(12.4%)	12	(13.0%)	0	(0%)	0.39

### Surgical data

The two groups showed no significant differences in operation time, the number of identified parathyroid glands, or reimplantation of parathyroid glands. However, the mean total weight of the excised thyroid gland in the Lugol group was observed to be non-significantly larger than that in the non-Lugol group, with weights of 42.2 g and 34.7 g, respectively. Unexpected discovery of papillary thyroid cancer was present in 5.1% of patients in the non-Lugol group, while no such finding was observed in the Lugol group (Table 2).

**Table 2:** Thyroidectomy data on patients diagnosed with Graves’ disease operated at Karolinska University Hospital between 2008 and 2015

		All (*n* = 813)		Non-Lugol treatment (*n* = 780)		Lugol treatment (*n* = 33)		*P* value
Operation time min mean (±SD)	All	105	( ± 30.4)	106	( ± 30.5)	102	( ± 26.9)	0.47
	Female	103	( ± 29.4)	103	( ± 29.5)	101	( ± 27.7)	0.62
	Male	121	( ± 33.1)	122	( ± 33.5)	107	( ± 23.4)	0.34
Number of identified parathyroid glands mean (±SD)	All	3.2	( ± 0.8)	3.2	( ± 0.8)	3.0	( ± 0.8)	0.13
	Female	3.2	( ± 0.8)	3.2	( ± 0.8)	2.9	( ± 0.8)	0.036
	Male	3.0	( ± 0.9)	3.0	( ± 0.9)	3.4	( ± 0.9)	0.30
Reimplantation of parathyroid gland n (%)	All	235	(28.9%)	228	(29.2%)	7	(21.2%)	0.32
	Female	211	(29.5%)	205	(29.8%)	6	(21.4%)	0.34
	Male	24	(24.7%)	23	(25.0%)	1	(20.0%)	0.80
Total excised thyroid gland weight g mean (±SD)	All	35.0	( ± 32.1)	34.7	( ± 32.1)	42.2	( ± 30.9)	0.065
	Female	33.1	( ± 27.4)	32.9	( ± 27.2)	40.0	( ± 30.4)	0.12
	Male	49.2	( ± 54.1)	48.9	( ± 55.1)	54.6	( ± 34.4)	0.45
Noted damage on recurrent laryngeal nerve peroperatively n (%)	All	20	(2.5%)	20	(2.6%)	0	(0%)	0.91
Indirect or direct laryngoscopy n (%)	Not performed	668	(84.2%)	640	(80.7%)	28	(84.9%)	0.68
	Normal	91	(11.5%)	86	(11.3%)	5	(15.2%)	0.46
	Paresis	34	(4.3%)	34	(3.1%)	0	(0%)	0.22
Re-bleeding with operation n (%)	All	9	(1.1%)	9	(1.1%)	0	(0%)	0.54
Unexpected papillary thyroid cancer post-operative n (%)	All	40	(4.7%)	40	(5.1%)	0	(0%)	0.18

### Calcium levels

No significant differences were observed in the mean levels of ionized calcium between the two groups before surgery or at the first follow-up visit, which took place between 4 and 6 weeks after the surgery. However, the Lugol group showed a slightly lower mean level of ionized calcium (1.12 mmol/L) compared to the non-Lugol group (1.15 mmol/L) on the first day postoperatively, but this was not statistically significant (Table 3).

**Table 3:** Pre- and post-thyroidectomy calcium levels in patients with Graves’ disease operated at Karolinska University Hospital between 2008 and 2015

	All (*n* = 813)		Non-Lugol treatment (*n* = 780)		Lugol treatment (*n* = 33)		*P* value
	Mean	SD	Mean	SD	Mean	SD	
Pre-operative							
Total calcium (mmol/L)	2.38	±0.17	2.38	±0.17	2.37	±0.11	0.76
Ionized calcium (mmol/L)	1.25	±0.10	1.25	±0.09	1.24	±0.06	0.74
Day 1 post-operatively							
Total calcium (mmol/L)	2.19	±0.21	2.19	±0.22	2.13	±0.18	0.21
Ionized calcium (mmol/L)	1.15	±0.11	1.15	±0.11	1.12	±0.09	0.21
First post-operative visit (4–6 weeks)							
Total calcium (mmol/L)	2.33	±0.15	2.33	±0.15	2.33	±0.15	1.00
Ionized calcium (mmol/L)	1.22	±0.08	1.22	±0.08	1.22	±0.08	0.98

Reference ranges for total calcium 2.15–2.50 mmol/L and ionized calcium 1.15–1.33 mmol/L

### Post-operative calcium and vitamin D supplements

At day 1 post-operatively 27.4% of all cases received oral calcium therapy. In the Lugol group 45.5% were treated with oral calcium compared to 26.7% in the non-Lugol group (*p* = 0.018). The relative risk for oral calcium treatment day 1 in the Lugol group was 1.70 (95% confidence interval (CI) 1.15–2.52, *p* = 0.018). No differences were seen in administration of intravenous calcium between the two groups.

The proportion of patients receiving calcium treatment at discharge was higher in the Lugol group compared to the non-Lugol group (36.4% vs 16.2%, *p* = 0.002). This indicates that the patients who received Lugol iodine before thyroid surgery had a relative risk of 2.25 (95% CI 1.39–3.63, *p* = 0.0025) to require calcium treatment at discharge than those who did not receive Lugol iodine. Additionally, vitamin D treatment for parathyroid insufficiency also differed between the two groups at discharge, with 36.4% of patients in the Lugol group receiving vitamin D treatment compared to 19.1% in the non-Lugol group (*p* = 0.015) (Table 4). The relative risk for vitamin D treatment at discharge in the Lugol group compared to the non-Lugol group was 1.90 (95% CI 1.19–3.06, *p* = 0.015).

**Table 4:** Medical treatment for hypocalcemia at day 1 postoperatively, discharge, first post-operative visit (4–6 weeks), and 4–6 months follow-up in patients diagnosed with Graves’ disease operated at Karolinska University Hospital between 2008 and 2015

		All (*n* = 813)		Non-Lugol treatment (*n* = 780)		Lugol treatment (*n* = 33)		*P* value
Day 1 post-operatively								
Hypocalcemia which has required oral treatment with calcium n (%)	All	223	(27.4%)	208	(26.7%)	15	(45.5%)	0.018
	Female	206	(28.8%)	193	(28.1%)	13	(46.4%)	0.035
	Male	17	(17.5%)	15	(16.3%)	2	(40.0%)	0.18
Hypocalcemia which has required i.v. treatment with calcium n (%)	All	25	(3.1%)	23	(3.0%)	2	(6.1%)	0.31
	Female	24	(3.4%)	22	(3.2%)	2	(7.1%)	0.26
	Male	1	(1.0%)	1	(1.1%)	0	(0%)	0.82
At discharge								
Hypocalcemia which has required treatment with calcium n (%)	All	138	(17.0%)	126	(16.2%)	12	(36.4%)	0.002
	Female	124	(17.3%)	113	(16.4%)	11	(39.3%)	0.002
	Male	14	(14.4%)	13	(14.1%)	1	(20.0%)	0.72
Vitamin D at discharge for parathyroid insufficiency n (%)	All	161	(19.8%)	149	(19.1%)	12	(36.4%)	0.015
	Female	149	(20.8%)	139	(20.2%)	10	(35.7%)	0.047
	Male	12	(12.4%)	10	(10.9%)	2	(40.0%)	0.054
First post-operative visit (4–6 weeks)								
Hypocalcemia which has required treatment with calcium n (%)	All	55	(6.8%)	52	(6.7%)	3	(9.1%)	0.75
	Female	46	(6.4%)	44	(6.4%)	2	(7.1%)	0.89
	Male	9	(9.2%)	8	(8.7%)	1	(20.0%)	0.67
	Missing	7	(0.9%)	7	(0.9%)	0	(0%)	
Vitamin D for parathyroid insufficiency n (%)	All	82	(10.1%)	75	(9.6%)	7	(21.2%)	0.082
	Female	71	(9.9%)	66	(9.6%)	5	(17.9%)	0.32
	Male	11	(11.3%)	9	(9.8%)	2	(40.0%)	0.11
	Missing	9	(1.1%)	9	(1.15%)	0	(0%)	
Post-operative visit (4–6 months)								
Hypocalcemia which has required treatment with oral calcium n (%)	All	19	(2.3%)	19	(2.4%)	0	(0%)	0.66
	Missing	22	(2.7%)	21	(2.7%)	1	(3.0%)	
Vitamin D for parathyroid insufficiency n (%)	All	24	(3.0%)	23	(3.0%)	1	(3.0%)	0.98
	Missing	30	(3.7%)	29	(3.7%)	1	(3.0%)	

At the first post-operative visit, which took place 4–6 weeks after surgery, calcium supplementation was reported in 6.8%, and vitamin D in 10.1% of all cases undergoing thyroidectomy, and there were no significant differences between the Lugol and non-Lugol groups (Table 4). At 4–6 months postoperatively, 2.3% of all cases were treated with calcium and 3.0% with vitamin D for hypoparathyroidism. No significant differences were observed between the Lugol and non-Lugol groups. No death or other serious complications were seen in either of the two groups.

## Discussion

Our main finding in this study was that the Lugol group showed an increased risk of requiring treatment for hypocalcemia compared to the non-Lugol group day 1 post-operatively and at discharge. The proportion of patients in the Lugol group receiving oral calcium treatment in the first day after surgery was 45.5% with a relative risk of 1.70. At discharge, the rates of calcium and vitamin D treatment, as a surrogate variable for hypocalcemia, were significantly higher in the Lugol groups than in the non-Lugol groups (relative risk 2.25 and 1.90, respectively).

There are several potential explanations for this phenomenon. One possible explanation is that the Lugol group may have had a shorter period of euthyroidism before surgery than the non-Lugol group, leading to a more significant manifestation of hungry-bone syndrome [14, 15]. Another possibility is that the surgical procedure is more complex in the Lugol group, potentially impacting the parathyroid glands to a greater extent [16]. However, it should be noted that the operation time remained unchanged between the groups, and the number of identified parathyroids was similar in both groups, which counters this explanation. While there was no statistically significant difference in thyroid weight, there was an indication suggesting a potential disparity. The Lugol group exhibited a mean thyroid weight of 42.2 g, whereas the non-Lugol group had a mean weight of 34.7 g. This finding could indicate a more challenging surgical procedure and its potential impact on parathyroid function.

Two recent retrospective studies on rescue versus elective thyroidectomy in Graves’ disease found no differences in temporary or permanent hypocalcemia/hypoparathyroidism and recurrent nerve palsy. In the study by Ali et al. there were 247 patients in the elective group [17]. The rescue group consisted of nineteen patients treated with Lugol iodine for ten days before surgery. In the study by Kartal et al. there were 101 cases in the elective group [18]. The rescue group consisted of twelve patients, nine were treated with Lugol iodine, and the remaining three received plasmapheresis. These studies had smaller sample sizes than our research, and type II errors cannot be ruled out.

The rate of post-operative hypocalcemia varies in the literature and can be attributed to different definitions and indications for surgery [19]. In a systematic review and meta-analysis including 115 observational studies, the median incidence of transient and permanent hypocalcemia was 27% (interquartile range (IQR) 19–38%) and 1% (IQR 0–3%), respectively [20]. These figures are in line with the results of all cases undergoing thyroidectomy in our study cohort.

Our study found no significant differences in patient characteristics or surgical data between the Lugol and non-Lugol groups except for a higher proportion of patients in the non-Lugol group being treated with anti-thyroid drugs and levothyroxine, as well as a higher proportion of patients in the Lugol group being treated with beta-blockers. The differences in the use of anti-thyroid drugs and beta-blockers between the Lugol and non-Lugol groups were expected considering the indications for the benefit of Lugol iodine in our setting.

We found papillary thyroid cancer in 5.1% of patients in the non-Lugol group, while no such finding was observed in the Lugol group. In a systematic review and meta-analysis of a cohort on incidental thyroid carcinoma in surgery-treated hyperthyroid patients with Graves’ disease, a pooled prevalence of 7.0% (95% CI 4.5% - 9.6%) was found [21]. This finding is in accordance with our results, although the differences in our study between the Lugol and non-Lugol groups pose a challenge to explain. However, it is important to note that the Lugol group consisted of only 33 patients, which could potentially influence the results due to the small sample size. Nevertheless, this observation stands out and may warrant further independent investigation.

The study has both strengths and limitations. The strengths include a large sample size, detailed patient characteristics, and surgical outcome information. However, the retrospective design limits the ability to establish causality and leaves room for potential confounding variables to influence the results. Furthermore, the study did not report long-term outcomes, such as persisting hypoparathyroidism beyond six months, which would be valuable in assessing complications in Graves’ disease-related thyroid surgery. Additional weaknesses include the limited number of Lugol patients, the absence of pre-surgery thyroid hormone levels and vitamin D status, and no information on anticoagulant treatment.

The register lacks details on length of post-operative stay in hospital. As we did not identify any variations in post-operative complications, such as bleeding, we did not specifically investigate this variable.

The register also lacks information on drainage. However, at Karolinska University Hospital drainage is primarily reserved for patients undergoing lateral neck lymph node dissection as part of thyroid cancer surgery. No information on intolerance, side effects or premature discontinuation of Lugol treatment are recorded in SQRTPA. Based on our cumulative clinical experience, patients typically encounter minimal difficulties in completing the medication with Lugol. The cost in Sweden for a bottle of Lugol’s solution containing 50 ml is approximately EUR 10.

Published data regarding side effects of potassium iodide are sparse. A field study in Poland after the Chernobyl accident estimated medically significant, but not serious, side effects in 0,2% after taking iodine blockade in one or more doses [22]. The reactions were headache, abdominal pain, diarrhea, vomiting, dyspnea, and skin rash. In a descriptive study on 27 patients with Graves’ disease treated with Lugol’s solution at Karolinska University hospital four patients (15%) reported limited adverse effects [12]. Moreover, an observational study on long term treatment with potassium iodide in 44 patients with Graves’ disease in Japan showed no side effects [23].

In this study we did not target patient satisfaction. Future studies should address this dimension for a comprehensive understanding.

In conclusion, patients with Graves’ disease who undergo thyroidectomy and are pre-operatively treated with Lugol iodine as a rescue therapy may have a higher risk of short-term hypocalcemia post-operatively, which warrants careful attention after surgery. However, this difference was not observed at follow-up. There were no significant differences between the two groups in terms of bleeding, pre- and post-operative calcium levels, laryngeal nerve damage, operation time, and thyroid weight. Thus, pre-operative treatment with Lugol iodine is safe.

## Data Availability

Restrictions apply to the availability of data generated or analyzed during this study to preserve patient confidentiality or because they were used under license. The corresponding author will on request detail the restrictions and any conditions under which access to some data may be provided.
